# The Complexity of the ERK/MAP-Kinase Pathway and the Treatment of Melanoma Skin Cancer

**DOI:** 10.3389/fcell.2016.00033

**Published:** 2016-04-27

**Authors:** Claudia Wellbrock, Imanol Arozarena

**Affiliations:** ^1^Manchester Cancer Research Centre, Wellcome Trust Centre for Cell-Matrix Research, The University of ManchesterManchester, UK; ^2^School of Applied Sciences, University of HuddersfieldHuddersfield, UK

**Keywords:** BRAF, MEK, ERK, melanoma, melanocytes, therapy, resistance, MITF

## Abstract

The central role played by the ERK/MAPK pathway downstream of RAS in human neoplasias is best exemplified in the context of melanoma skin cancer. Signaling through the MAPK pathway is crucial for the proliferation of melanocytes, the healthy pigment cells that give rise to melanoma. However, hyper-activation of the MAPK-pathway is found in over 90% of melanomas with approximately 50% of all patients displaying mutations in the kinase BRAF, and approximately 28% of all patients harboring mutations in the MAPK-pathway up-stream regulator NRAS. This finding has led to the development of BRAF and MEK inhibitors whose application in the clinic has shown unprecedented survival responses. Unfortunately the responses to MAPK pathway inhibitors are transient with most patients progressing within a year and a median progression free survival of 7–10 months. The disease progression is due to the development of drug-resistance based on various mechanisms, many of them involving a rewiring of the MAPK pathway. In this article we will review the complexity of MAPK signaling in melanocytic cells as well as the mechanisms of action of different MAPK-pathway inhibitors and their correlation with clinical response. We will reflect on mechanisms of innate and acquired resistance that limit patient's response, with a focus on the MAPK signaling network. Because of the resurgence of antibody-based immune-therapies there is a growing feeling of failure in the targeted therapy camp. However, recent studies have revealed new windows of therapeutic opportunity for melanoma sufferers treated with drugs targeting the MAPK pathway, and these opportunities will be discussed.

## The ERK/MAP-kinase pathway is a crucial regulator of melanocyte proliferation and differentiation

**Figure 1:**
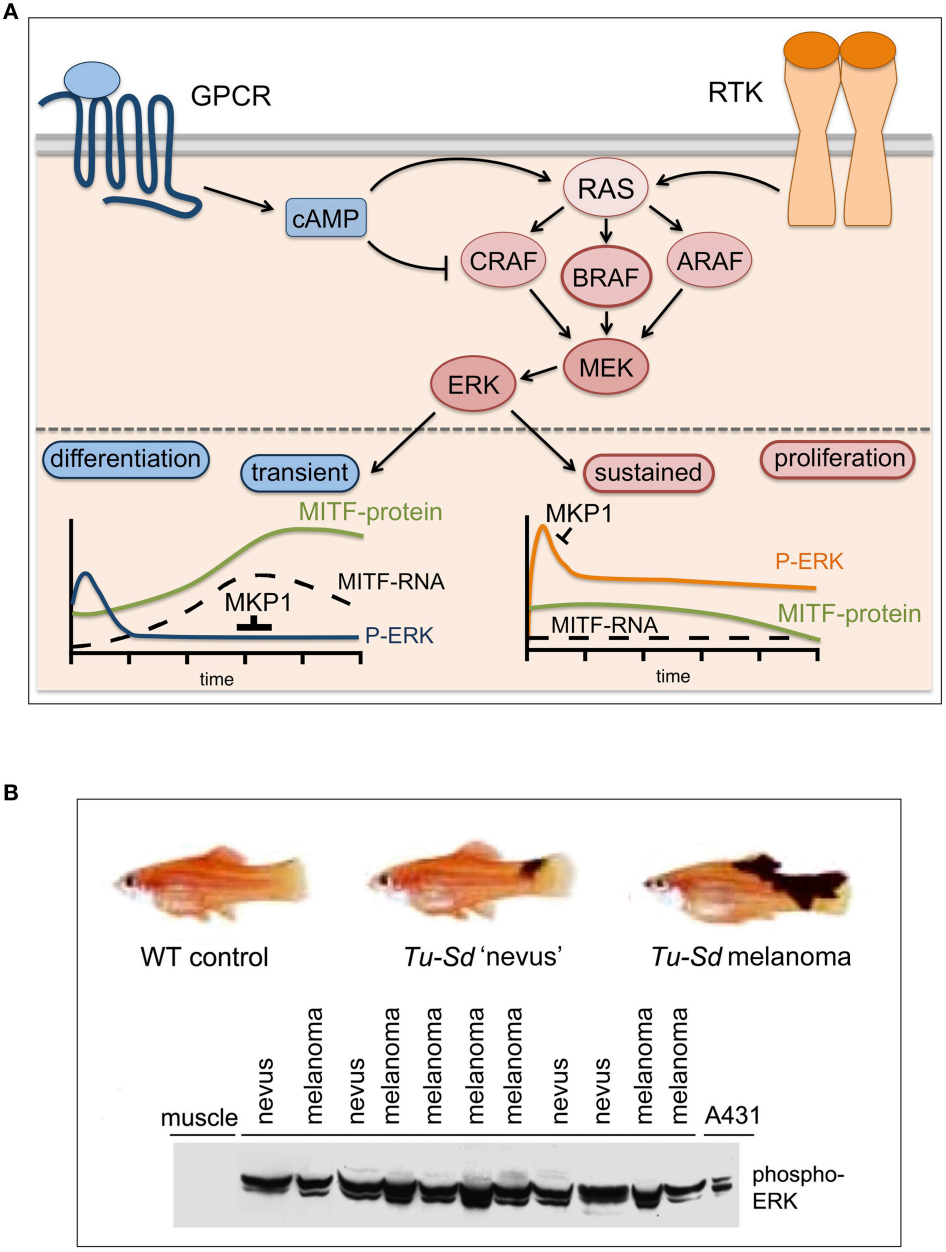
**MAPK pathway signaling: transient, sustained, and constitutive. (A)** In normal melanocytes the ERK/MAPK pathway is governed by G-protein couple receptor (GPCR) induced cAMP signaling (i.e., MC1R) and receptor tyrosine kinase (RTK) signaling. MC1R signals mainly through BRAF, while CRAF is inhibited (Busca et al., 2000; Dumaz et al., 2006). All receptors lead to ERK activation, but through the differential induction of the ERK phosphatase DUSP1 (MKP1) this results in either transient or sustained ERK activation (Wellbrock et al., 2002b). The melanocyte fate regulator MITF is an ERK target and sustained ERK phosphorylation stimulates its degradation (Wu et al., 2000), which keeps MITF levels low- a state competitive with proliferation. However, cAMP signaling induces MITF expression and in the absence of active ERK this leads to MITF up-regulation, which triggers differentiation. **(B)** In *Xiphophorus* hybrids harboring the macromelanophore locus *Tu-Sd* (Tumor-spotted dorsal pattern) in which melanoma development is driven by the melanocyte specific overexpression of the EGFR homolog Xmrk, ERK is constitutively activated in early benign “nevus”-like lesions and its activation is maintained in malignant melanomas. A phospho-ERK Western blot of lysates (100 μg total protein) from individual fish carrying either nevi (*n* = 4) or malignant melanomas (*n* = 7) is shown. Lysates from A431 cancer cells and from muscle tissue served as positive and negative control respectively (adapted from Wellbrock and Schartl, 1999).

Cutaneous melanoma originates from melanocytes, neural-crest derived pigment-producing cells located in the epidermis, where their major function is to protect keratinocytes from UV-induced DNA damage (Abdel-Malek et al., 2010). Under basal conditions and in response to UV the physiology of a melanocyte is modulated by keratinocytes, which secret specific paracrine acting factors (Hirobe, 2011). These secreted factors stimulate a broad spectrum of intracellular signaling. However, a crucial downstream event triggered by almost all of the extracellular factors is the activation of the ERK/MAP-kinase (MAPK)-pathway, which plays a major role in coordinating the balance between melanocyte differentiation and proliferation (see Figure 1A).

Up-regulation of intracellular cAMP levels, which induces the differentiation process (Busca and Ballotti, 2000), triggers a very transient (≤ 60 min) and weak activation of ERK (Wellbrock et al., 2002b). On the other hand, activation of the MAPK-pathway by the synergistic action of factors like SCF, FGF, or HGF stimulates strong sustained ERK activation, which triggers melanocyte proliferation (Bohm et al., 1995).

At the center of this transient vs. sustained ERK activation is MITF (Figure 1A), a tissue specific bHLH-Zip transcription factor and fate regulator of the melanocyte lineage, which is a target of ERK phosphorylation (Hemesath et al., 1998; Figure 1A). MITF regulates the expression of genes controlling differentiation (e.g., *TYR*) proliferation (e.g., *CDK2*) and survival (e.g., *BCL2, BCL2A1*) (Wellbrock and Arozarena, 2015). As ERK phosphorylation can increase MITF's transcriptional activity toward *TYR* (Hemesath et al., 1998), transient ERK activation can favor differentiation, and in the context of cAMP signaling this is paralleled by a strong transcriptional up-regulation of the MITF transcript (Price et al., 1998). However, ERK phosphorylation can also trigger ubiquitin-mediated degradation (Wu et al., 2000), and as a result of sustained ERK activation MITF protein levels are reduced, a situation that is compatible with proliferation (Wellbrock and Marais, 2005). Nevertheless, because MITF is crucial for cell survival, its expression in proliferative cells is ensured through the ERK induced transcription factor BRN2 (Wellbrock et al., 2008). In summary, the MAPK-pathway has stringent control over the melanocyte/melanoma fate-decision regulator MITF, which might explain why this pathway is so particularly critical in the biology of a melanocytic cell and hence in melanoma.

## The discovery of the relevance of ERK/MAP-kinase signaling for melanoma

Melanoma is not one of the cancers with the highest incidences when compared to breast, lung or colon cancer and therefore historically not much attention was given to the research directed toward a better understanding of this skin cancer. However, this changed dramatically in 2002, when the Cancer Genome Project/Sanger Institute identified oncogenic mutations in the MEK-upstream kinase *BRAF* in over 50% of melanoma (Davies et al., 2002). This discovery led to an explosion in published work on the relevance of the MAPK-pathway in melanoma; as such research into melanoma can be divided in the pre- and post-2002 era.

The development of the MEK inhibitors PD908059 and U0126 in the pre-2002 era resulted in the first studies demonstrating a role for MEK in human melanoma cell proliferation, survival and invasion (Kortylewski et al., 2001; Li et al., 2001). The first indication for an *in vivo* relevance of MAPK signaling in this disease came however from Xiphophorus, a genetically controlled vertebrate model for melanoma first described in 1928 (Wellbrock et al., 2002a). In these animals strong constitutive MAPK activation occurs already in benign nevus-like lesions (Wellbrock and Schartl, 1999), suggesting an involvement of MAPK-signaling in the early steps of pigment-cell transformation (Figure 1B). In 2002, Cohen et al. reported constitutive ERK-phosphorylation in >20% of benign nevi and >80% of primary melanoma, and hence confirmed activation of MAPK-signaling as an early event in human melanoma development (Cohen et al., 2002).

Since the first description of *BRAF* mutations in melanoma (Davies et al., 2002) BRAF^V600E^, the most predominant mutant, has been shown to constitutively activate ERK in melanocytes, and to transform *p16/INK4A* deficient melanocytes (Wellbrock et al., 2004). BRAF^*V*600*E*^ induces melanoma in mice, where this can be accelerated by the absence of *p16/INK4A* or the PI3K-antagonist *PTEN*, or by UV exposure (Dankort et al., 2009; Dhomen et al., 2009; Viros et al., 2014). In line with what has been observed in humans, in zebrafish BRAF^V600E^ only triggers the formation of benign nevi (Patton et al., 2005). However, in zebrafish mutants where a temperature shift lowers levels of functional MITF (possibly compatible with proliferation), BRAF^V600E^ efficiently induces melanoma (Lister et al., 2014; Zeng et al., 2015). This further emphasizes the relevance of the BRAF/MITF connection for melanoma development.

In humans, BRAF^V600E^ mutations are found in benign nevi (Pollock et al., 2003), clonal populations of senescent melanocytes (Gray-Schopfer et al., 2006). BRAF^V600E^ can stimulate senescence in human melanocytes *in vitro* (Michaloglou et al., 2005). Hence, nevi might represent the result of oncogene-induced senescence. Nevertheless, formation of a nevus requires an initial pulse of melanocyte proliferation, and MAPK signaling appears to be essential for this step. This has been very elegantly shown in zebrafish that develop invasive melanoma induced by mutant RAS, which is however completely abolished when RAS is rendered incapable of activating MAPK signaling (Michailidou et al., 2009). Importantly, these fish do not even develop nevi, clearly demonstrating that no melanocyte proliferation had occurred in the absence of constitutive MAPK signaling, and tumor initiation was completely blocked (Michailidou et al., 2009).

During the last 14 years numerous studies have established the relevance of BRAF^V600E^-induced MAPK signaling for most aspects of human melanoma development and progression; this includes proliferation, survival, hypoxia, invasion, and angiogenesis (Huntington et al., 2004; Karasarides et al., 2004; Gaggioli et al., 2007; Kumar et al., 2007; Klein et al., 2008; Johansson et al., 2009).

## The complexity of ERK/MAP-kinase signaling in melanoma

The relevance of the MAPK-pathway for melanoma is reflected in the overall rate of mutations leading to deregulation of the pathway. These include not only the ~50% of BRAF mutations, but also >25% *NRAS* mutations and ~14% of melanomas with mutations in the RAS suppressor *NF1* (CancerGenomeAtlasNetwork 2015).

In contrast to *BRAF*, mutations in the other isoforms, *CRAF* and *ARAF* are rare. This is thought to be due to the more complex mechanisms underlying activation of these isoforms (Emuss et al., 2005). As a consequence activation of ARAF or CRAF would require at least two mutation events, while the BRAF kinase can be rendered active by one mutation event. The majority of these mutations affect the phosphate-binding loop (P-loop) or the activation loop (A-loop) in the kinase domain (Davies et al., 2002). The most common V600E substitution mimics phosphorylation of the A-loop, inducing an active conformation of the kinase (Wan et al., 2004; Garnett et al., 2005).

Surprisingly, other mutations were found to render BRAF inactive (Davies et al., 2002). The biochemical analysis of these “kinase-impaired” mutations revealed that although they reduce BRAF's enzymatic activity, BRAF still activates MEK through dimerization with CRAF in a RAS dependent manner (Wan et al., 2004; Garnett et al., 2005). Confirming earlier studies (Weber et al., 2001), it is now well established that RAF kinases homo and hetero-dimerize partly in a RAS dependent manner. Importantly, these interactions can impact on the response to inhibitors of BRAF. Thereby, inhibitor-binding triggers dimerization and in the presence of (hyper)-active RAS, instead of pathway-inhibition, this leads to the so called “paradoxical” pathway-activation through CRAF (Wan et al., 2004; Garnett et al., 2005; Hatzivassiliou et al., 2010; Heidorn et al., 2010; Poulikakos et al., 2011). Elucidation of this complex mechanism has proven valuable in the understanding of some of the side effects that BRAF inhibitors produce in patients (see below).

The MAPK-pathway is not linear but part of a complex network containing scaffold proteins and feedback loops. The scaffold protein KSR competes with CRAF for inhibitor-induced BRAF-binding and can counteract the “paradoxical activation” of ERK (McKay et al., 2011), but another scaffold protein, IQGAP promotes ERK activation and the targeted interruption of its interaction with ERK1/2 can contribute to MAPK-pathway inhibition (Jameson et al., 2013). Furthermore, complex feedback loops are induced through the expression of phosphatases (e.g., DUSP6) or adaptor proteins (e.g., SPROUTY) (Hanafusa et al., 2002; Owens and Keyse, 2007). This is also crucial in the context of BRAF inhibition in patients, where these negative feedback mechanisms are relieved with the subsequent up-regulation of other MEK up-stream regulators allowing MAPK pathway activation without BRAF involvement (Lito et al., 2013).

## The development of BRAF, MEK, and ERK inhibitors

First attempts to inhibit BRAF^V600E^ in patients using sorafenib (BAY 43-9006), a broadband kinase inhibitor originally designed to inhibit CRAF, were rather disappointing (Eisen et al., 2006). However, between 2011 and 2014 the FDA and the EMA have approved the use of vemurafenib (PLX4032) and dabrafenib (GSK2118436) for the treatment of BRAF mutant melanoma patients. Both, vemurafenib and dabrafenib bind to the active site in the kinase domain in its “DGF-in” (active) conformation, thereby blocking the access to ATP, and both inhibitors display similar potency for BRAF^V600E^ and CRAF and selectivity against many other kinases (Bollag et al., 2010; Waizenegger et al., 2016). Phase I to III trials using these drugs showed impressive, unprecedented clinical responses in the field of targeted therapies with overall responses of 80%, median progression free survival between 6 and 9 months and median overall survival rates between 13 and 19 months (Flaherty et al., 2010; Chapman et al., 2011; Hauschild et al., 2012; Long et al., 2012; Sosman et al., 2012). Of note, up to 30% of patients treated with BRAF inhibitors develop RAS driven cancers such as squamous cell carcinomas, colon cancer or leukemia (Flaherty et al., 2010; Chapman et al., 2011; Callahan et al., 2012). These “side-effects” are most likely due to the “paradoxical” activation of CRAF in RAF dimers upon inhibitor-binding to wild-type BRAF. As mentioned before, paradoxical activation of CRAF depends on active RAS and is thus favored in cells that signal through RAS (Hatzivassiliou et al., 2010; Heidorn et al., 2010; Poulikakos et al., 2011).

In parallel, inhibitors targeting MEK (e.g., selumetinib, trametinib, cobimetinib) have been developed. BRAF's unique effector is MEK, and pre-clinical studies have shown that BRAF mutant cells are significantly more sensitive to MEK inhibition inhibitors than RAS mutant cells (Solit et al., 2006), probably due to RAS activating other pathways such as the PI3K-cascade to promote cell survival (Haass et al., 2008). Despite drug related toxicities limiting the use of MEK inhibitors, recently developed highly potent inhibitors show efficacy in patients (Flaherty et al., 2012; Kirkwood et al., 2012; Ascierto et al., 2013).

Recently, the attention has also moved to ERK and the first ERK inhibitors that are effective in both, BRAF and NRAS mutant as well as cells that have developed resistance to MEK inhibitors have been described (Hatzivassiliou et al., 2012; Morris et al., 2013). Trials testing SCH772984 and GDC-0994 are currently ongoing.

## Mechanisms of resistance to BRAF and MEK inhibitors

Despite the outstanding responses obtained with BRAF inhibitors, in the majority of patients clinical responses are transient. The analysis of melanomas from patients relapsed on BRAF inhibitor treatment revealed the vast complexity of the MAPK signaling network and over the last years a plethora of mechanisms have been identified that allow cells to bypass BRAF inhibition by activating other signaling nodes eventually re-establishing MEK activity and hence reactivation of ERK [for a detailed review see Lito et al., 2013], which is thought to occur in >70% of patients (Shi et al., 2014; Van Allen et al., 2014).

**Figure 2:**
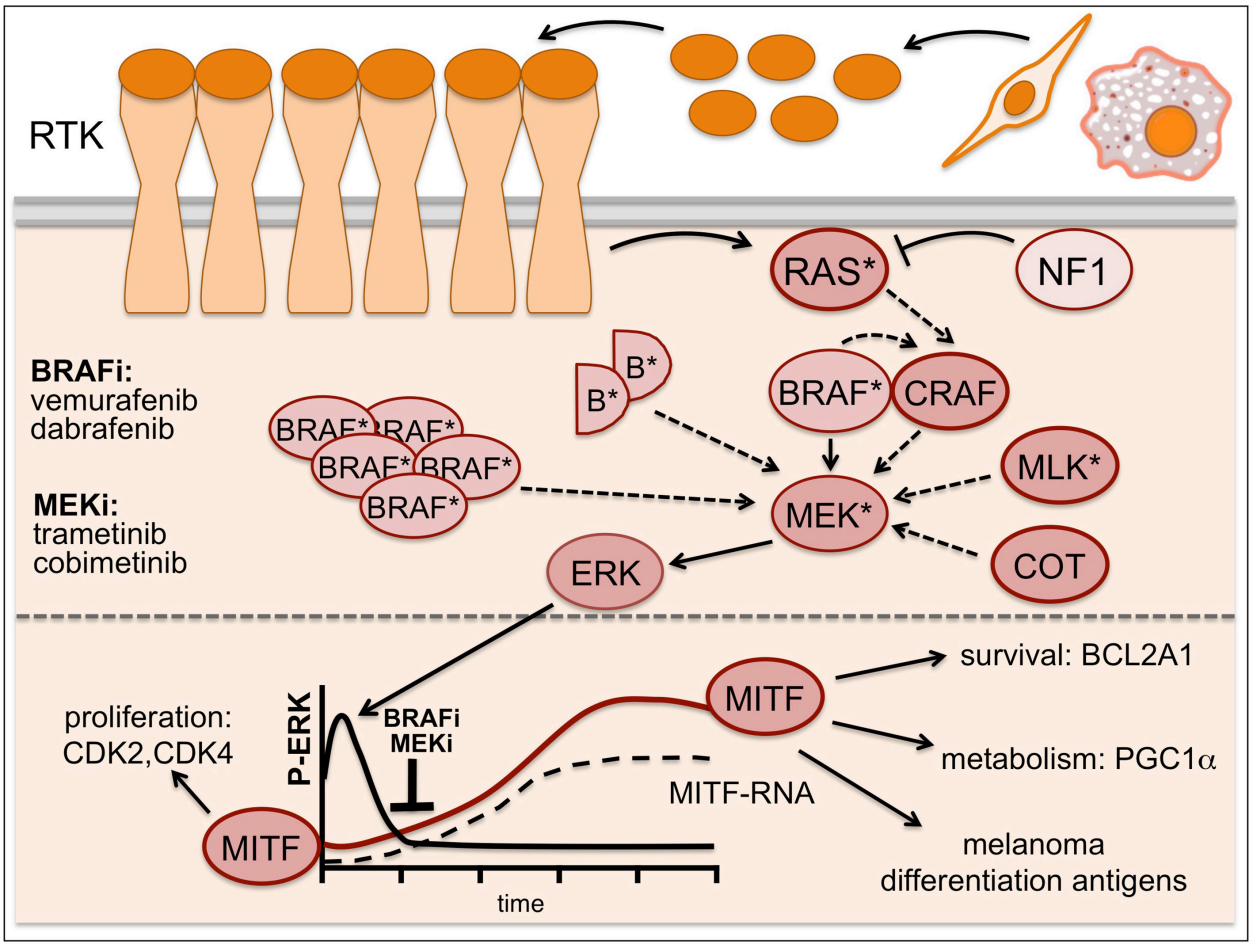
**Resistance mechanisms in MAPK-inhibitor treated melanoma**. Resistance to BRAF inhibitors can occur through activating *NRAS* mutations (^*^), loss of the RAS suppressor *NF1, BRAF* amplification or alternative splicing leading to BRAF truncations (semicircles B^*^), overexpression or mutation of the MEK activators CRAF, COT/TPL2/MAP3K8 or MLKs as well as MEK mutations (^*^). Addition of a MEK inhibitor can buffer some of these resistance mechanisms, but eventually enhanced resistance signaling will overcome its effects. While ERK is inhibited -particularly in the initial phases of treatment- MITF expression is up-regulated and contributes to drug-tolerance. Solid arrows indicate signaling induced by mutant BRAF, dashed arrows indicate signaling brought about by the various resistance mechanisms.

Some of these mechanisms (Figure 2) involve activating *NRAS* mutations or loss of the RAS suppressor *NF1* (Whittaker et al., 2013), *BRAF* amplification or alternative splicing leading to BRAF truncations (Poulikakos et al., 2011; Shi et al., 2012) and overexpression or mutation of the MEK activators CRAF, COT/TPL2/MAP3K8 or MLKs (Montagut et al., 2008; Johannessen et al., 2010; Marusiak et al., 2014). BRAF inhibitor action can also be overcome by mutations in MEK itself, and while some of these mutations increase the basal kinase activity of MEK, others render the kinase insensitive to MEK inhibitors (Emery et al., 2009; Wagle et al., 2011).

Intriguingly, increased receptor tyrosine kinase (RTK) signaling through for instance IGF-1R, PDGFR, or EGFR is also frequently found in relapsed melanomas (Nazarian et al., 2010; Villanueva et al., 2010; Girotti et al., 2013; Sun et al., 2014), and this can lead to ERK activation via classical pathway activation through RAS and CRAF (Figure 2). Moreover, RTK signaling has been linked to an intrinsically BRAF inhibitor resistant phenotype (Konieczkowski et al., 2014; Muller et al., 2014), which was unexpected, as RTK signaling was not perceived as being a major driver of human melanoma. Indeed, it appears that in heterogeneous tumors RTK-signaling melanoma cells are present with lower frequency. However, this balance changes in the presence of a BRAF inhibitor, when RTK-signaling becomes advantageous (Sun et al., 2014). That RTKs in fact can be very potent drivers of melanoma development is seen in Xiphophorus hybrids, where overexpression of an EGFR homolog stimulates proliferation (Wellbrock et al., 1998) and is sufficient to initiate and progress melanoma development (see Figure 1B).

Another BRAF/MEK inhibitor resistance mechanism is based on overexpression of pro-survival factors that allow melanoma cells to evade apoptosis even under complete/efficient ERK inhibition. Around 30% of melanomas display amplifications in the *BLC2A1* gene (Haq et al., 2013). Over-expression of the anti-apoptotic BLC2A1 protein blocks BRAF and MEK inhibitor induced apoptosis, and intriguingly, BCL2A1 expression is regulated by MITF (Haq et al., 2013; Figure 2). This together with other target genes might underlie the fact that MITF itself can confer resistance to BRAF and MEK inhibitors even when ERK is not re-activated (Smith et al., 2013; Muller et al., 2014). This becomes relevant on the initial phase of treatment, where (in line with low ERK activity being correlated with increased MITF levels; see Figure 1A) the majority of patients show significant up-regulation of MITF as early response (Figure 2). Importantly, this increased MITF expression can contribute to drug-tolerance in the initial phases of treatment (Smith et al., 2016).

Apart from cell-autonomous resistance, the tumor-stroma can also confer resistance to BRAF inhibitors (Figure 2). HGF secreted by stromal fibroblasts can circumvent BRAF inhibition by re-activating ERK through cMET/RAS/CRAF-signaling (Straussman et al., 2012) and stromal fibroblasts can alter the ECM and produce resistance by engaging integrin/FAK signaling (Hirata et al., 2015). Furthermore, tumor associated macrophages can induce resistance via the secretion of VEGF or TNFα (Smith et al., 2014; Wang et al., 2015). Secreted factors can also support the outgrowth of innate resistant cells that are otherwise slow cycling (Obenauf et al., 2015).

The above-described examples reflect the complexity of inhibiting the MAPK-pathway as therapy strategy, because interfering with this central pathway *in vivo* will inevitably have also an effect on non-cancer cells. As a consequence, it can be expected that the entire tumor microenvironment will readjust to the condition of reduced MAPK signaling and establish a new balance that eventually can “buffer” the drug effect.

## The future of MAPK-pathway targeting drugs in melanoma

Resistance through BRAF-inhibitor bypass and the development of RAS-driven secondary cancers in responses to BRAF inhibition have prompted the development of combination therapies with BRAF and MEK inhibitors. These combinations prolong responses and significantly reduce the appearance of RAS-driven secondary malignancies, but unfortunately patients still develop resistance (Larkin et al., 2014; Long et al., 2015). Nevertheless, BRAF/MEK inhibitor combinations are now accepted as the standard of care for BRAF-mutant advanced melanoma and the trametinib/dabrafenib and cobimetinib/vemurafenib combinations received FDA approval in 2014 and 2015, respectively. In addition, drugs targeting both BRAF and CRAF and interfering with dimerization have been described (Girotti et al., 2015; Yao et al., 2015), but whether the use of such inhibitors might increase systemic toxicity will have to be assessed.

Currently, great effort is put into developing novel combination strategies to conquer resistance and prolong responses, and one of the main combination targets for such an approach is the PI3-kinase pathway (PI3-kinase, mTOR, AKT). The reason for this is its central role in melanoma, which is reflected in its frequent deregulation through mutations (CancerGenomeAtlasNetwork 2015), and these are found even more frequently in BRAF/MEK inhibitor resistant tumors (Shi et al., 2014; Van Allen et al., 2014). Furthermore, PI3-kinase signaling is also activated downstream of mutant *NRAS* suggesting possible MEK/PI3-kinase inhibitor combinations. While pre-clinical studies provide strong evidence for the rationale of these combinations, the latest clinical trials show that these combinations are poorly tolerated and toxicity limits efficacy (Bedard et al., 2015; Tolcher et al., 2015). Nevertheless, as the PI3-kinase pathway is central to many cancers the aim is to identify the crucial -and possibly cancer-specific- nodes within the pathway and design more specific and potent inhibitors (Kwong and Davies, 2013).

Other combinations (e.g., with RTK-inhibitors) are currently trialed, and of course it is considered to combine MAPK-pathway inhibitors with immunotherapies. However, the toxicities observed in the first attempts demonstrate that we require a much better understanding of the role of MAPK-signaling in the context of immunity.

## Conclusions

Over the last 5 years the use of MAPK inhibitors in melanoma patients and the development of resistance to these drugs has revealed the vast complexity of MAPK signaling that occurs in a multicellular organism. However, while the ability of the MAPK pathway to rewire has so far played against its inhibition, there might be an opportunity to take advantage of this and target the rewiring. As such, pre-clinical studies support the concept of a drug-holiday, where drugs are administered intermittently to break the rewiring (Das Thakur et al., 2013). Another possibility is to directly target the “rewired phase.” In this phase, in which cells display an almost uniform rewiring response and >80% of tumors react with MITF up-regulation, targeting the rewiring-mechanism produces impressive responses in pre-clinical studies (Smith et al., 2016) Thus, with all the excitement about the latest immunotherapy successes, it should not be forgotten that BRAF and MEK inhibitors produce immediate and impressive results and long-lasting (>4 years) responses are also observed in a number of melanoma patients (Puzanov et al., 2015). This clearly demonstrates that there is room for further improvement that will allow building on the remarkable achievements of these targeted therapies.

## Author contributions

CW and IA contributed to the writing of the article.

## Funding

Work in the laboratory of CW is funded by Cancer Research UK (grant C11591/A16416).

### Conflict of interest statement

The authors declare that the research was conducted in the absence of any commercial or financial relationships that could be construed as a potential conflict of interest.
